# Beta blocker use in subjects with type 2 diabetes mellitus and systolic heart failure does not worsen glycaemic control

**DOI:** 10.1186/1475-2840-11-14

**Published:** 2012-02-14

**Authors:** Bryan Wai, Leighton G Kearney, David L Hare, Michelle Ord, Louise M Burrell, Piyush M Srivastava

**Affiliations:** 1 Department of Cardiology, Austin Health, Heidelberg, VIC, Australia; 2 Department of Medicine, University of Melbourne, Austin Health, Heidelberg, VIC, Australia

**Keywords:** Beta-blockers, Diabetes, Systolic heart failure, Glycaemic control

## Abstract

**Background:**

The prognostic benefits of beta-blockers (BB) in patients with systolic heart failure (SHF) are known but despite this, in patients with diabetes they are underutilized. The aim of this study was to assess the effect of beta-blockers (BB) on glycaemic control in patients with Type 2 Diabetes (T2DM) and systolic heart failure (SHF) stratified to beta-1 selective (Bisoprolol) vs. nonselective BB (Carvedilol).

**Methods:**

This observational, cohort study was conducted in patients with T2DM and SHF attending an Australian tertiary teaching hospital's heart failure services. The primary endpoint was glycaemic control measured by glycosylated haemoglobin (HbA1c) at initiation and top dose of BB. Secondary endpoints included microalbuminuria, changes in lipid profile and estimated glomerular filtration rate (eGFR).

**Results:**

125 patients were assessed. Both groups were well matched for gender, NYHA class and use of guideline validated heart failure and diabetic medications. The mean treatment duration was 1.9 ± 1.1 years with carvedilol and 1.4 ± 1.0 years with bisoprolol (*p *= ns). The carvedilol group achieved a reduction in HbA1c (7.8 ± 0.21% to 7.3 ± 0.17%, *p *= 0.02) whereas the bisoprolol group showed no change in HbA1c (7.0 ± 0.20% to 6.9 ± 0.23%, *p *= 0.92). There was no significant difference in the change in HbA1c from baseline to peak BB dose in the carvedilol group compared to the bisoprolol group. There was a similar deterioration in eGFR, but no significant changes in lipid profile or microalbuminuria in both groups (*p *= ns).

**Conclusion:**

BB use did not worsen glycaemic control, lipid profile or albuminuria status in subjects with SHF and T2DM. Carvedilol significantly improved glycemic control in subjects with SHF and T2DM and this improvement was non significantly better than that obtained with bisoprolol. BB's should not be withheld from patients with T2DM and SHF.

## Background

The prognostic benefits of beta-blockers (BB) in patients with systolic heart failure (SHF) are known [1,2] but despite this, patients with diabetes have been identified as receiving suboptimal treatment with BB [3,4]. The prevalence of SHF in patients with T2DM is ~ 12% whilst in patients with left ventricular systolic dysfunction 6-25% have T2DM [5]. It would seem clear that in the management of patients with both T2DM and SHF, use of beta-blockers whilst maintaining good glycaemic control is paramount to improved clinical outcomes [6-8]. In hypertensive subjects with T2DM without SHF, carvedilol has been shown to have favorable effects on glycaemic control in comparison with metoprolol tartrate [9].

We aimed to assess the glycaemic control of patients with T2DM and SHF treated with BB in a tertiary teaching hospital and the differential effects of a nonselective BB (carvedilol) versus a β1 selective BB (bisoprolol) on glycaemic control, renal function, albuminuria and lipid profile.

## Methods

### Patients

Consecutive patients that were referred following an index hospitalization with decompensated SHF and T2DM to our multidisciplinary heart failure clinic were enrolled. Patients were followed up prospectively.

### Heart failure management

Patients received either carvedilol or bisoprolol and the doses were titrated to a maximal tolerated dose (target of 10 mg of bisoprolol or 50 mg of carvedilol per day). The choice of beta-blocker was left to the discretion of the treating cardiologist, with other heart failure management utilization as per accepted guidelines [2]. Patients included were not on beta-blockers prior to index hospitalization.

### Diabetes management

Patients were managed for their diabetes by their primary care and specialist diabetes physician. The number of anti-diabetic medications in both groups during the follow-up period did not change.

### Measured variables

SHF was defined as presence of symptoms and signs of heart failure and left ventricular ejection fraction less than 50%. New York Heart Association Class (NYHA) was recorded at the first outpatient visit along with collection of serum and urine samples at commencement and within 3 months of achieving peak tolerated dose of BB. Glycaemic control was assessed by glycosylated haemoglobin (HbA1c) which is measured by automated HPLC (Bio-Rad Laboratories, California, USA). Renal function by estimated Glomerular Filtration Rate (eGFR) and albuminuria by using the ratio of urinary albumin concentration to urinary creatinine concentration (ACR). Microalbuminuria was defined as ACR greater than 30 mg/g and less than 300 mg/g. To assess changes in lipid profile, fasting total cholesterol (TC), high-density lipoprotein (HDL) and low-density lipoprotein (LDL) and triglyceride (TG) level were measured according to previously published methods [10].

### Statistical analysis

Continuous data are presented as mean ± standard deviation and categorical data as n (%). Changes in HbA1c, eGFR, microalbuminuria and lipid profile were examined using t-tests. Categorical variables were compared using Fisher's exact test. Statistical significance was taken as *p *< 0.05.

## Results

Data from a total of 125 patients with SHF and T2DM was analyzed (n = 80 carvedilol, n = 45 bisoprolol). The mean treatment duration from baseline to peak BB dose was 1.9 ± 1.1 years with carvedilol and 1.4 ± 1.0 years with bisoprolol (*p *= ns). The mean peak dose of carvedilol was 26.5 ± 21.1 mg/day and bisoprolol was 5.8 ± 3.0 mg/day. Both groups were well matched for gender (majority male), NYHA class, and use of guideline validated therapies i.e. renin angiotensin system inhibitors, diuretics, spironolactone and diabetes treatment (diet, oral hypoglycaemics and/or insulin). (Table 1)

**Table 1:** Patient's baseline characteristics

	Carvedilol (n = 80)	Bisoprolol (n = 45)	*p*-value
**Demographics**			
Male	62 (78%)	34 (76%)	0.83
Age (years)	71.0 ± 9.7	70.7 ± 10.8	0.89
NYHA (mean)	2.1 ± 0.7	2.1 ± 0.6	0.90
NYHA Class I	14 (18%)	5 (11%)	
NYHA Class II	45 (56%)	30 (67%)	
NYHA Class III	18 (22%)	10 (22%)	
NYHA Class IV	3 (4%)	0	
**Diabetic Medications**			
Diet	12 (15%)	5 (11%)	0.60
Oral hypoglycaemic	49 (61%)	27 (60%)	1.00
Insulin	19 (24%)	13 (29%)	0.53
**Heart Failure Medications**			
ACEI	61 (76%)	33 (73%)	0.83
ARB	24 (30%)	14 (31%)	1.00
ACE I and ARB	9 (11%)	4 (9%)	0.77
Spironolactone	50 (62%)	26 (58%)	0.70
Thiazide	11 (14%)	9 (20%)	0.45

ACEI = Angiotensin Converting Enzyme Inhibitor, ARB = Angiotensin Receptor Blocker

**Figure 1:**
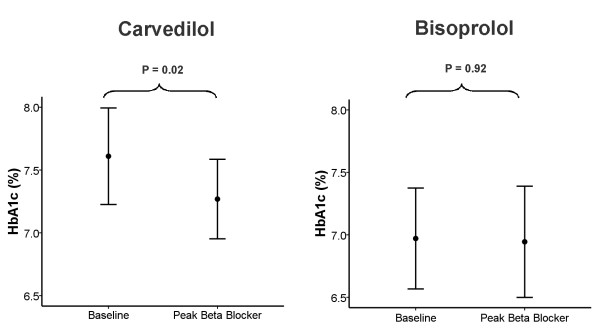
**Mean HbA1c ± standard deviation at baseline and at peak beta-blocker dose**.

For the primary endpoint, glycaemic control improved in the carvedilol group but no significant change was noted in the bisoprolol group (Table 2). No significant difference was seen between the changes in HbA1c in the carvedilol group vs. bisoprolol group (-0.5 ± 1.4% vs. 0.2 ± 1.3%; *p *= 0.09) (Figure 1). Both groups had significant reductions in eGFR from baseline to peak BB dose, but with no significant difference in this reduction between the carvedilol and bisoprolol groups. The proportion of patients with microalbuminuria remained the same in both groups for the duration of the study. There were no significant differences in the lipid profile between the two groups for the duration of the study.

**Table 2:** Glycaemic control, lipid profile and renal function in both groups at baseline and at peak beta-blocker dose

	Carvedilol			Bisoprolol		
	Baseline	Peak BB	*p*-value	Baseline	Peak BB	*p*-value
Glycaemic control						
HbA1c	7.7 ± 1.5	7.2 ± 1.2	0.02	7.0 ± 1.2	6.9 ± 1.3	0.92
Lipid Profile						
TC/HDL	4.1 ± 1.0	3.7 ± 1.1	0.07	3.1 ± 1.0	3.0 ± 1.2	0.67
LDL/HDL	2.2 ± 0.9	2.0 ± 0.9	0.08	1.5 ± 0.7	1.5 ± 1.0	0.83
TG	1.9 ± 1.2	2.0 ± 1.5	0.66	1.6 ± 1.2	1.5 ± 0.9	0.52
Renal function						
eGFR	53.6 ± 27.8	48.4 ± 27.4	0.01	60.4 ± 24.2	51.1 ± 26.9	< 0.01
ACR	0.7 ± 0.8	0.6 ± 0.72	0.6	0.9 ± 0.9	0.7 ± 1.0	1.00

## Discussion

The major finding of this study is that BB use did not worsen glycaemic control, lipid profile nor albuminuria status in patients with T2DM and SHF, suggesting that these medications should not be withheld in this high-risk group. This is in contradiction to the GEMINI study, where hypertensive T2DM patients randomized to carvedilol (nonselective BB) did not have a significant change in HbA1c whereas patients on metoprolol tartrate (selective β1 BB) had a significant increase in HbA1c [9]. Futhermore, in a sub-analysis of the GEMINI study an increase in insulin resistance as measured by homeostasis model assessment-insulin resistance (HOMA-IR) was found in patients treated with metoprolol compared to carvedilol[11]. In another study, metoprolol use in patients with type 2 diabetes mellitus was shown to be associated with a significant reduction in insulin-stimulated endothelial function where as this function was preserved with carvedilol use[12] One proposed mechanism for this was that selective β1 BB's such as atenolol and metoprolol may cause vasoconstriction, decreased peripheral blood flow and may exacerbate insulin resistance [13], whereas the α-adrenergic blocking effect of carvedilol may allow greater peripheral blood flow and hence increased utilization of glucose. . This differential effect on glycaemic control seen in hypertensive diabetics may not be applicable to patients with systolic heart failure due to their overactive sympathetic tone. In addition we found that there was a significant improvement in glycaemic control between baseline to peak BB dose within the carvedilol group but not within the bisoprolol group. Whilst there was also a strong trend for better glycaemic control in the carvedilol group compared to the bisoprolol group, this did not quite reach statistical significance. This result could possibly have been biased by different baseline HbA1c levels between the two groups and therefore does not provide absolute evidence for the differential effects on glycaemic control between carvedilol and bisoprolol.

The GEMINI study also showed that patients on carvedilol had a greater reduction in microalbuminuria when compared to patients taking metoprolol [14]. Such differences were not seen in our study. This could be explained by the fact that there is a known higher incidence of microalbuminuria in patients with SHF [15]. Half of the patients in our study had microalbuminuria for the duration of the study, which is a higher proportion than found in the general population of patients with T2DM. The National Health and Nutrition Examination Survey found microalbuminuria in 29% of patients with diabetes mellitus [16] whereas the PREVEND study noted only 16% of patients with diabetes mellitus had microalbuminuria [17]. The higher incidence of microalbuminuria in our study is likely to be mediated via impairment of endothelial function[15,18]. A recent study by Jawa et al. in African American subjects with T2DM and hypertension suggested that there was an improvement in endothelial function and albuminuria with commencement of carvedilol but without significant change in albuminuria with metoprolol [19]. In contrast, a recent study in in patients with mild heart failure (16% diabetic) failed to show a change in endothelium-dependent vasodilatation when the beta-blocker was changed from carvedilol to metoprolol succinate or tartrate [20]. We did not find any significant changes in albuminuria over the follow-up period in either group.

A concern of treating physicians has often been that BB use would lead to elevation of triglycerides and lowering of HDL. A study by Pollare et al. demonstrated significant elevation in LDL to HDL ratio and triglyceride levels with the use of metoprolol or atenolol [21]. In a more contemporary cohort, beta-blocker use was not associated with a deterioration in lipid profile but suggest greater statin use with metoprolol when compared to carvedilol[22]. In our study, no worsening in lipid profiles was seen in either group of patients whilst on BB. This might be related to a greater use of statins amongst our cohort of patients.

Traditional teaching for T2DM has been that BB may worsen hypoglycaemic awareness, glycaemic control and lipid metabolism and should be used with caution. However, the prognosis of patients that develop SHF is poor and is markedly improved by appropriate BB therapy. Although the use of beta-blockers in T2DM and SHF has not been specifically studied, meta-analyses of large-scale clinical trials have demonstrated the prognostic benefit of beta-blockers in SHF patients who also have diabetes mellitus [23]. Indeed to the authors knowledge there is only one other study that has compared carvedilol to bisoprolol in patients with SHF where, retrospectively after 18 months of follow up, no significant differences were found in survival or cardiac morbidity [24] In addition, strict glycaemic control is essential to the management of patients with diabetes mellitus. In the United Kingdom Prospective Diabetic Study, lowering of HbA1c toward 6% resulted in significant reductions of micro vascular and macro vascular complications and death related to T2DM, including SHF [8]. Furthermore, poor glycaemic control is associated with increased incidence of heart failure, hospitalization and death [7]. It is clear that in the management of patients with T2DM and SHF, the use of beta-blockers whilst maintaining good glycaemic control is important.

## Limitations

This study was an observational cohort study and not a randomised control trial. Hence the choice of beta blocker and diabetic therapies were left to the treating cardiologist and endocrinologist and thus could be a possible source of bias. Despite this, both cohorts were well matched at baseline for diabetes and heart failure treatment. Nevertheless, because the initial HbA1c readings were different between the two groups, it is difficult to draw an absolute conclusion regarding the differential effects of carvedilol and metoprolol. (selective vs. nonselective)

## Conclusion

In conclusion, BB use did not worsen glycaemic control, lipid profile nor albuminuria status in patients with T2DM and SHF, suggesting that these medications should not be withheld in this group of patients. Carvedilol significantly improved glycemic control in patients with T2DM and SHF and that this improvement was non significantly better than that obtained with bisoprolol.

## Abbreviations

BB: Beta blocker; SHF: Systolic heart failure; T2DM: Type II diabetes mellitus; HbA1c: Glycosylated haemoglobin; eGFR: Estimated glomerular filtration rate; NYHA: New York heart association; ACR: Albumin creatinine ratio; TC: Total cholesterol; HDL: High density lipoprotein; LDL: Low density lipoprotein; TG: Triglycerides.

## Competing interests

The authors declare that they have no competing interests.

## Authors' contributions

BW, LGK, MO, PMS: Conception, data acquisition, analysis, drafting and revising manuscript. DLH, LMB: Data acqusition, analysis, drafting and revising manuscript. All authors read and approved the final manuscript.
